# Influence of Community-Led Total Sanitation and Water Coverages in the Control of Cholera in Madarounfa, Niger (2018)

**DOI:** 10.3389/fpubh.2021.643079

**Published:** 2021-04-29

**Authors:** Julien Graveleau, Maria Eleanor Reserva, Alama Keita, Roberto Molinari, Guillaume Constantin De Magny

**Affiliations:** ^1^West and Central Africa Regional Office, UNICEF, Dakar, Senegal; ^2^Health Systems Strengthening Unit-Health Section, West and Central Africa Regional Office, UNICEF, Dakar, Senegal; ^3^Niger Country Office, UNICEF, Niamey, Niger; ^4^Department of Mathematics and Statistics, Auburn University, Auburn, AL, United States; ^5^Centre for Research on the Ecology and Evolution of Diseases (CREES), Montpellier, France; ^6^MIVEGEC (Université de Montpellier, UMR CNRS 5290, IRD 229), Institut de Recherche pour le Développement Délégation Occitanie, Montpellier, France

**Keywords:** cholera, WASH, Africa, hurdle model, odd-ratio

## Abstract

Every year, cholera affects 1.3–4.0 million people worldwide with a particularly high presence in Africa. Based on recent studies, effective targeting interventions in hotspots could eliminate up to 50% of cases in Sub-Saharan Africa. Those interventions include Water, Sanitation, and Hygiene (WASH) programs whose influence on cholera control, up to the present, has been poorly quantified. Among the few studies available, D'Mello-Guyett et al. underline how the distribution of hygiene kits is a promising form of intervention for cholera control and that the integration of a WASH intervention at the point of admission of suspected cases is new in cholera control efforts, particularly in outbreaks and complex emergencies. Considering the limited number of studies on Community-Led Total Sanitation (CLTS) and water coverages related to cholera control, the aim of our work is to determine whether these interventions in cholera hotspots (geographic areas vulnerable to disease transmission) have significant impact on cholera transmission. In this study, we consider data collected on 125 villages of the Madarounfa district (Niger) during the 2018 cholera outbreak. Using a hurdle model, our findings show that full access to improved sanitation significantly decreases the likelihood of cholera by 91% (*P* < 0.0001) compared to villages with no access to sanitation at all. Considering only the villages affected by cholera in the studied area, cholera cases decrease by a factor of 4.3 in those villages where there is partial access to at least quality water sources, while full access to improved water sources decreases the cholera cases by a factor of 6.3 when compared to villages without access to water (*P* < 0.001). In addition, villages without access to safe water and sanitation are 6.7 times (*P* < 0.0001) more likely to get cholera. Alternatively, villages with full sanitation and water coverage are 9.1 (*P* < 0.0001) less likely to get cholera. The findings of our study suggest that significant access to improved water and sanitation at the village level offer a strong barrier against cholera transmission. However, it requires full CLTS coverage of the village to observe a strong impact on cholera, as partial access only has a limited impact.

## Introduction

The relationship between cholera and contaminated drinking water was first established by John Snow in the mid-19th century (1, 2). Following these findings, access to safe water and improved sanitation in high-income countries over the past two centuries has definitively eliminated cholera transmission of toxigenic *Vibrio cholerae* (3). Despite this progress, the seventh pandemic of cholera is still ongoing since the 1960s, particularly in Sub-Saharan African countries where the majority of the burden is usually reported with the exception of unprecedented cholera outbreaks like that in Haiti between 2010 and 2019 (4) or Yemen since 2016 (5). Basic safe water and sanitation access remains a challenge (58 and 36% coverage, respectively) (6).

The fact that waterborne diseases that are transmitted through the fecal–oral route, like diarrheal diseases, has been largely studied, leading to guidelines and standards (7–10). Among the programs that can be implemented at the community level to improve sanitation, Community-Led Total Sanitation (CLTS) is an innovative methodology for mobilizing communities to completely eliminate open defecation (OD). Communities are facilitated to conduct their own appraisal and analysis of OD and take their own action to become open defecation-free (ODF). The result of becoming ODF is usually the construction of toilets, latrines that avoid fecal matter directly contaminating the environment.

Based on recent studies, effective targeting interventions in hotspots could eliminate up to 50% of cases in Sub-Saharan Africa (11). Among the few studies available, D'Mello-Guyett et al. (12) underline how the distribution of hygiene kits is a promising form of intervention for cholera control and that the integration of a WASH intervention at the point of admission of suspected cases is new in cholera control efforts, particularly in outbreaks and complex emergencies (12). Even though the relationship between cholera and Water, Sanitation, and Hygiene (WASH) has long been established (13), the beneficial impact of water and sanitation projects like CLTS, specifically in cholera hotspots, is often challenged due to the lack of evidence and the complexity in demonstrating such an impact (e.g., long-term implementation of WASH projects, availability of data, sustainability of WASH projects) (14). Questioning has increased over the past years, as oral cholera vaccines (OCVs) have shown short- to medium-term impacts on cholera (15), creating a tendency to promote medical solutions over WASH solutions whose benefits, among others, are not limited to their impact on cholera. Aside from mapping the outbreak of cholera under the guidelines of the Millennium Development Goals (MDGs) and the Sustainable Development Goals (SDGs) of the United Nations (16), the few existing references on cholera control either exclusively focus on the use of OCVs (17, 18) or on their joint usage with WASH interventions (19, 20). To the best of our knowledge, only one study highlights the use of hygiene kit distribution as a promising intervention for cholera control, particularly in outbreaks and complex emergencies (12).

For all these reasons, this work intends to present the results of a study carried out for the Madarounfa district (Niger), which was classified as a hotspot according to the World Health Organization Global Cholera Task Force definition (21), concentrating over the time period between 1994 and 2017, recording 13 outbreaks with an average duration of 14 weeks (22). The most recent cholera outbreak occurred in 2018, officially resulting in 2,628 cases and 42 deaths. Indeed, over the past few years, WASH projects that include CLTS have been implemented in Madarounfa in order to create ODF communities, improve hygiene practices, and demand access to safe drinking water. These conditions allowed to perform a cross-sectional study collecting data on population and its health and sanitation conditions from 125 villages in the district. Among these villages, 14 did not have access to sanitation or water, 79 had partial access to both water and/or sanitation, and 32 had full sanitation and water coverage. Combining this information with other socio-environmental factors (see further on), this study therefore seeks to determine if differences exist in the burden of cholera between villages of the Madarounfa district based on their water and CLTS coverages while taking into account other available influential factors.

## Materials and Methods

### Data Sources

We collected all available data on cholera, past or present WASH programs, population census, and socio-environmental information at the village level for Madarounfa district (Niger) for the year 2018 over different sources (Internet, public reports available locally, and professionals from the Health district and Hydraulic and Sanitation district). Usually, data are compiled at a national or provincial level. In the case of this study, most of the data were collected directly at the district level for village level type of information and had to be compiled by ourselves. The sources of data included the Madarounfa district report on CLTS for 2018; line-listing of cholera cases from health centers of the Health District of Madarounfa in 2018; REF2017-REGMI database from the “Ministry of Hydraulic and Sanitation” providing information on water infrastructures, water coverage, and GPS data; MADA-POP-2017-25CSI providing population data from 2017 based on projection from last census; Community Development Plans (CDP-2018–2022) from Gabi, Safo, and Sarkin Yamma providing socio-environmental information. Finally, distances between villages and between surface water and villages were measured directly through the dedicated tools proposed by the Google Earth website to see if these factors could be associated with cholera transmission.

The data were collected in December 2018 in Madarounfa district from the Maradi Province. Field visits in the studied areas were also carried out between December 2018 and April 2019 to cross-check information through observational methods.

Finally, other sources of information such as JMP 2018 (23) or “Niger cholera factsheet” (from Regional Cholera Platform^1^) was used to compare the area of study to the national context.

### Community Led Total Sanitation Context

CLTS aims to create ODF certification, which means that 100% of the households have at least basic access to sanitation and have hand-washing facilities. Based on data collected from the 125 studied villages, 53 villages (i.e., 42%) have been certified in recent years, with 100% of the village households reporting sanitation and hand-washing facilities. Some villages were not yet involved in CLTS projects, as 40% of the villages had no access to sanitation (which means no household of this village had access to basic sanitation). Only 22 villages (i.e., 18%) had partial access to sanitation (ranging from 29 to 97% of CLTS involvement coverage in those villages), as they either were still involved in CLTS projects or did not complete the CLTS project. Villages were classified based on three levels of CLTS coverage determined as follows: (1) villages with no access to sanitation (0% coverage); (2) villages with partial access (ranging from 1 to 99% of the household with latrine and hand-washing facility) to sanitation; (3) villages with full CLTS coverage (100% of the household with latrine and hand-washing facility).

In the studied area, the main sources of unsafe water are rivers, lakes, dams, or open wells. Functioning hand pumps and tap stands (from small water networks with deep boreholes) were considered basic drinking water sources. Basic drinking water services are defined as drinking water from an improved source (piped water, boreholes or tube wells, protected dug wells, protected springs) according to the WHO/UNICEF Joint Monitoring Program (JMP) for Water Supply, Sanitation, and Hygiene, provided collection time is not more than 30 min for a round trip and number of users <500 persons per water point.

Water-related data are reported in Table 1, summarizing the number of cholera cases, number of villages, and total inhabitants as a contingency table with two entries. The first entry is the aggregation of these data with the water coverage categorized into three levels: (1) Villages with no access to basic water services representing 24 villages (i.e., 19% of the sample of villages); (2) Villages with partial access to basic water services (36 villages with water coverage ranging from 33 to 94%). For this level, villages with more than 500 persons per basic water source and villages with both safe and unsafe water sources were considered; (3) Villages with full basic water services coverage (65 villages representing 52% of the studied villages), which refer to all villages with maximum 500 persons per safe water point and no alternative unsafe water source. The second entry is the aggregation of these data with CLTS coverage categorized into three levels: (1) Villages with no sanitation coverage representing 50 villages (i.e., 40% of the sample of villages); (2) Villages with partial sanitation coverage (22 villages with sanitation coverage ranging from 29 to 97%); (3) Villages with full sanitation coverage (53 villages representing 42.4% of the studied villages).

**Table 1:** Number of villages, inhabitants, and cholera cases based on their basic water access levels and Community-Led Total Sanitation (CLTS) coverage levels.

	**Villages without access to basic water**			**Villages with partial access to basic water**			**Villages with at least full basic water access**		
	**# of villages**	**Inhabitants**	**Cholera**	**# of villages**	**Inhabitants**	**Cholera**	**# of villages**	**Inhabitants**	**Cholera**
			**cases**			**cases**			**cases**
Villages without sanitation coverage	14	5,435	81	16	24,058	73	20	41,425	147
Villages with partial sanitation coverage	1	898	0	8	8,99	46	13	16,073	13
Villages with full sanitation coverage	9	4,483	7	12	11,578	1	32	22,741	8

# *Abbreviation of number*.

With 52% of the studied villages having access to basic water coverage and 42% having access to basic sanitation, the studied area is above Niger's standards but remains representative of the national picture. In fact, based on JMP (2017) (6), only 50% of the people of Niger have access to basic water services and 14% have access to basic sanitation.

### Village Stratification

The study sample collects data on 125 villages including 41 villages from the Gabi Commune, 26 villages from the Safo Commune, and 58 from the Sarkin Yamma Commune for a total population targeted by the study of 135,680 persons in 2018. The last census in Safo reports 79,024 inhabitants, 101,704 inhabitants in Gabi, and 44,704 inhabitants in Sarkin Yamma, as 60.2% of the population of those three targeted communes were considered due to lack of data in some villages. Three communes over the five existing ones of Madarounfa district were included in this study.

### Data Compilation for the Study

A final database was created for the study compiling all the available data for each village therefore including the following variables: (1) number of inhabitants; (2) number of cholera cases from the 2018 outbreak; (3) prevalence of cholera per 1,000 inhabitants from the 2018 outbreak; (4) water coverage at the village level; (5) sanitation coverage per village; (6) distance between each village and the nearest cholera-affected village; (7) distance between a village and the nearest surface water; (8) availability of road access to the village.

With the exact GPS location of the 125 studied villages (from REF2017-REGMI database of the “Ministry of Hydraulic and Sanitation”), it was possible to measure the distances between villages and the nearest affected villages as well as the nearest surface water points (often a river). Road access to the village was also captured through satellite imagery as a potential influencing factor on the burden of cholera per village.

### Data Analysis

In this study, we choose to analyze the data according to two methodologies, the odds ratios (ORs) as a statistic that quantifies the strength of the association between two events and commonly used in epidemiology and a statistical class model motivated by an excess of zeros in the data, the hurdle model. Such statistical approaches on CLTS effects on cholera epidemiology are aimed to detail evidence on the degree of CLTS involvement of the population to sustainably impact the transmission of cholera.

#### Odds Ratios

ORs were calculated to quantify the association of CLTS and water coverages to the prevalence of cholera in order to identify any correlation. Since OR measures the association but does not quantify the impact of sanitation and water coverages and the prevalence of cholera, a hurdle model was used to test for these possible relationships.

#### Hurdle Model

The reason for choosing a hurdle model was to account for the numerous zero cholera cases (77 villages out of 125) and because we were interested in understanding the factors that discriminate between the presence or not of cholera, on one side, and the factors that are associated with reducing the prevalence of cholera if observed, on the other side. Based on these choices, we chose a binomial distribution to model the presence or absence of cholera, while a negative binomial distribution was used for the positive cases since a Poisson model did not account for the overdispersion (24). This kind of model is particularly adapted for count data and is used in many applications such as biology, epidemiology, and public health (25, 26). In order to deliver numerically stable results, we projected the prevalence rate of each village onto a standardized population of 10,000 people under the assumption that this would not greatly affect the final statistical tests. By doing so, we do not need to use weights in the modeling process.

To test the influence of CLTS interventions on the prevalence of cholera, we used a three-level factor variable for access to improved water source (none, partial, or full access to improved water source). A three-level factor variable was also used for sanitation (none, partial, or full access to improved sanitation). Concurrently, we also added non-WASH variables, namely, distance to the nearest open-water source (in meters), distance of village to the nearest contaminated village (in meters), and access to dust road as explanatory factors.

For the hurdle model analysis, we first fit a full hurdle model with all the explanatory variables (Model 1). Then, we ran two models each with WASH and non-WASH factors only (Model 2 and Model 3, respectively). For both water and sanitation factor variables, we used no water access and no sanitation as the base level.

A log-likelihood test was performed by comparing the fit of the full hurdle model to the fit of WASH and non-WASH models. By removing predictor variables from a model, the model will likely fit less well with a lower log likelihood, but it is necessary to test whether the observed difference in model fit is statistically significant. Having log-likelihood results that are statistically significant will conclude that the less restrictive model (i.e., full hurdle model) is said to fit the data significantly better than the more restrictive model (i.e., WASH or non-WASH models).

## Results

### General Information on the Studied Villages

The Community Development Plans of the studied areas revealed that they are constituted of 92% Haoussa people, 5% Peulhs, 1% Touaregs, and 2% coming from other tribes, with 99% of the overall population being Muslim (27–29). All communes report eight children on average per woman and around nine persons per household. Children under 15 years old represent 54% of the population. From an economic point of view, all the villages mention agriculture as the main source of livelihood and animal breeding as the secondary source of income. Climatic factors such as rainfall (max: 686 mm/year to min: 498 mm/year) and temperature (between 40°C in April/May to 18°C in January/February) are similar between villages.

The dunes and sandy areas represent about half of the area studied, and it is used for seasonal agriculture during the rainy season. The valleys represent one quarter of the area of the three communes where most of the population lives and practices irrigation in small vegetable gardens (+300 m altitude as the lowest point of the studied area). The remaining area is made of bushes used from grassing in higher and rocky land (+514 m as the highest point of the studied area).

Finally, it is important to notice that the area targeted for the study has never benefited from a cholera vaccination campaign. We have concluded to a similarity between the communes and villages studied.

### Incidence of Cholera

**Figure 1:**
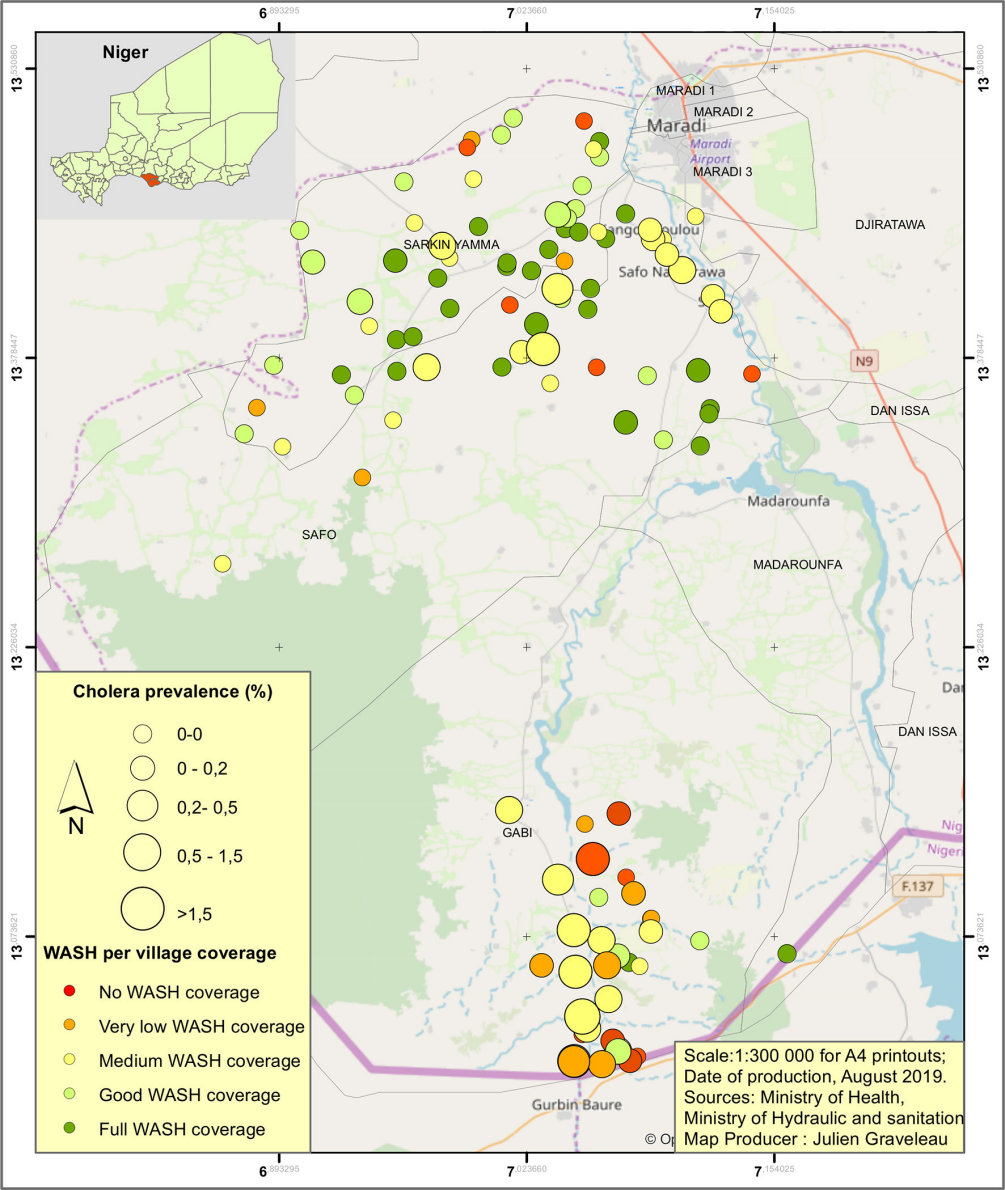
Map of the prevalence of cholera in the studied villages during the 2018 cholera outbreak regarding the Water, Sanitation, and Hygiene (WASH) coverage.

The incidence of cholera in villages with respect to the level of WASH coverage is illustrated in Figure 1. It shows clearly that cholera incidence is negatively related to the level of WASH coverage, with higher incidence in the villages located in the south of the area where most of the WASH coverage is between medium to no WASH coverage.

The distribution of cholera cases, inhabitants, and number of villages regarding both the basic water access and the access to sanitation levels are presented in Table 1.

### Odds Ratios

**Figure 2:**
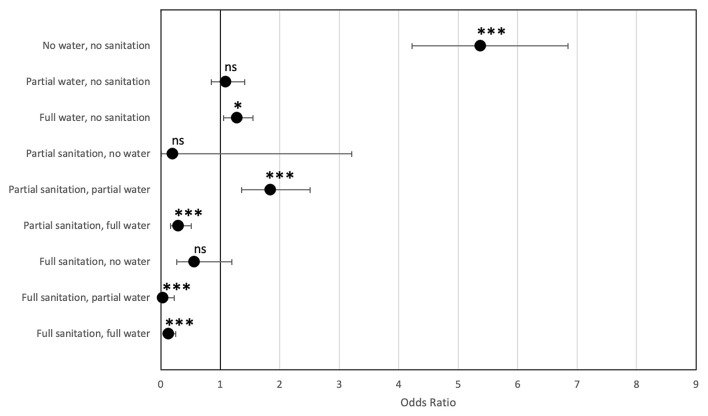
Odds ratios of cholera prevalence according to water and sanitation coverage (95% confidence interval). Z statistics for the odds ratio (Null hypothesis H_0_ odds ratio = 1) ns, non-significant, *P < 0.05, ***P < 0.001.

Using OR, strong links between CLTS and water coverages and prevalence of cholera were detected. Findings show that villages without access to safe water and sanitation are 5.38 times more likely to get cholera (95% confidence interval from 4.22 to 6.85; *P* < 0.0001). Alternatively, villages with full sanitation and water coverage are 7.88 less likely to get cholera (OR 0.13; 95% confidence interval 0.06–0.26; *P* < 0.0001) (Figure 2).

Figure 2 shows that if partial access to safe water already has an impact on the risk of cholera (compared to no access), it seems that full CLTS coverage (capturing both sanitation and hand-washing facilities) is required at the village level to observe a significant impact. However, CLTS (sanitation and hygiene) has a stronger impact on cholera than only water access, as we observe that prevalence remains high at 1.28 (95% confidence interval from 1.05 to 1.55; *P* < 0.0001) for villages with full water coverage but no sanitation. Once full CLTS is reached, prevalence of cholera drops significantly regardless of water coverage of the village.

In the dataset, the prevalence rate of cholera was 14.9 per 1,000 inhabitants in villages without water or sanitation (81 cases of cholera over 5,435 inhabitants), and only 0.4 per 1,000 inhabitants in villages with full access to sanitation and water (eight cases over 22,741 inhabitants).

### Hurdle Models

The log-likelihood test results detailed in Table 2 determined that the WASH-only model (Model 2) shows the best goodness-of-fit.

**Table 2:** The log-likelihood test results between full Hurdle Model vs. Water, Sanitation, and Hygiene (WASH) only (model 1 vs. model 2) or vs. Non-WASH (model 1 vs. model 3).

**Model**	**Log likelihood**
Full Hurdle Model vs. WASH only (Model 1 vs. Model 2)	−309.55
Full Hurdle Model vs. Non-WASH (Model 1 vs. Model 3)	−325.59^***^

*** *P < 0.0001*.

In terms of its contribution to the likelihood of cholera cases, only fully improved sanitation level shows significant contribution. Compared to the base case of having no access to improved sanitation at all, full access to improved sanitation decreases the likelihood of cholera by 91%, resulting as significant at a 99% confidence level (*P* < 0.01). Water access, both full and partial, is not significant to cholera cases at this level. None of the non-WASH factors, namely, distance to the nearest open-water source (in meters), distance of village to the nearest contaminated village (in meters), and access to dust road as explanatory factors, is significant (Figure 1).

When cholera cases actually occur, access to improved water sources corresponds to a decrease in cases of cholera. Cholera cases decrease by a factor of 4.354 where partial access to improved water source is observed, while full access to improved water source decreases it by a factor of 6.315. This is significant at a 99% confidence level (*P* < 0.01). Full sanitation corresponds to a decrease of cholera cases by a factor for 4.923 times compared to having no access to improved sanitation. Non-WASH factors are statistically equivalent to zero. The results are similar when running WASH-only and non-WASH-only specifications (Figure 2).

One village of interest (Village Kabobi) has a disproportionate high prevalence of cholera compared to the rest of the villages (30). While not having a considerable impact in terms of conclusions (see Supplementary Material 1), we chose to exclude this village from the analysis given the possible effects that outliers can have when performing statistical estimation and testing (30). In this sense, we therefore preferred to underestimate the impact of CLTS on cholera outbreaks rather than the contrary (Table 3). With this in mind, the analysis (without the outlier) showed that full sanitation access has significant impact both when considering the likelihood of observing cholera cases and when having a relation with case magnitude when cholera cases are actually observed. The likelihood of cholera cases decreases by 91%, significant at a 99% confidence level (*P* < 0.01), with full sanitation access. Partial sanitation access decreases the likelihood by 78%, significant at a 95% confidence level (*P* < 0.05). When cholera cases are actually observed, cases decrease by a factor of 0.3 when there is full access to sanitation, which is lower, but still significant at a 95% confidence level (*P* < 0.05), compared to the analysis with Village Kabobi. Non-WASH factors considered in our models remain non-significant (Supplementary Material 1).

**Table 3:** Full and partial [Water, Sanitation, and Hygiene (WASH) and non-WASH] hurdle models with village Kabobi.

**Variables**	**Model (1)**		**Model (2)**	
	**Coefficients**	**Transformed^#^**	**Coefficients**	**Transformed^#^**
**COUNT MODEL**				
Water Access–Partial	−1.471^**^ (0.520)	−4.354^**^	−1.429^**^ (0.524)	0.239^**^
Water access–Full	−1.843^***^ (0.500)	−6.315^***^	−1.696^***^ (0.463)	0.183^***^
Sanitation–Partial	0.518 (0.439)	0.518	0.521 (0.449)	1.683
Sanitation–Full	−1.594^***^ (0.434)	−4.923^***^	−1.547^***^ (0.442)	0.213^***^
Distance to Water	0.000016 (0.0000349)	1.000		
Distance to contaminated village	NA (NA)	NA		
Road access	0.246 (0.388)	0.246		
Log Theta	0.092 (0.13)	0.092	−0.118 (0.228)	
Intercept	5.342^***^ (0.395)	5.342^***^	5.555^***^ (0.432)	258.485^***^
**ZERO HURDLE MODEL**				
Water access–Partial	1.133 (0.668)	0.756	1.062 (0.647)	0.743
Water access–Full	1.281 (0.653)	0.782	1.168 (0.616)	0.763
Sanitation—Partial	−1.306 (0.6004)	0.213	−1.442 (0.567)	0.191
Sanitation–Full	−2.298^***^ (0.628)	0.091^***^	−2.526^***^ (0.519)	0.074^***^
Distance to water	−0.000042 (0.000163)	0.499		
Distance to contaminated village	−0.0000841 (0.000215)	0.484		
Road access	0.0606 (0.513)	0.499		
Intercept	0.178 (0.665)	0.544	−0.204 (0.499)	

*Standard errors in parentheses*.

*** *P < 0.0001,*

** *P < 0.01*.

# *Zero–part coefficients uses of logit link function; plogis() function was applied to transform the coefficients. The count-part coefficients were transformed via exponentiation*.

## Discussion

In this study, we aimed to test the hypothesis of a significant association of the water and CLTS coverages on the burden of cholera during the 2018 cholera outbreak in Madarounfa, Niger. Both employed statistical methods point to a significant decrease of the burden of cholera with an increase of safe water access and CLTS coverages. OR results showed that villages without access to safe water and sanitation are 6.66 times (95% confidence interval from 5.2 to 8.53; *P* < 0.0001) more likely to get cholera. Alternatively, villages with full sanitation and full water coverages are 9.1 less likely to get cholera (OR 0.11; 95% confidence interval 0.05–0.22; *P* < 0.0001).

Using a hurdle model on the 125 villages, findings show that full access to improved sanitation and hygiene significantly decreases the likelihood of observing cholera by 91% (*P* < 0.0001) compared to the villages with no access to improved sanitation at all.

Considering villages affected by cholera within the area of study, partial access to improved water sources decreases the cases of cholera by a factor of 4.3, while full access to improved water decreases the number of cholera cases by a factor of 6.3 compared to villages without access to water.

We did not find any other significant factors such as distance to nearest contaminated villages, distance to nearest surface water, or road access to have an influence on the burden of cholera at the village level.

Our results on cholera are consistent with the findings of discussing the positive health impacts of sanitation on diarrhea, even when the only water available was unimproved (8). In addition, water improvements did not result in a reduction of cholera if sanitation remained unimproved, as shown for the health impacts of diarrhea (8). As for diarrhea, we showed that synergy of improvements in water and sanitation together is producing larger impacts than the case when implemented in the absence of the order, specifically in the rural context of our study.

Focusing more specifically on previous studies about the association between water, sanitation, and hygiene exposures and cholera, Wolfe et al. (13) in their systematic review and meta-analysis unexpectedly found that no sanitation factor or improved water source (with the exception of bottled water) was significantly protective against cholera. In this study, they do not doubt the effectiveness of these interventions, confirming that the risk factors are consistently risky, and that factors that are expected to interrupt cholera transmission indeed have the potential to do so but are not always effective due to the complexity of the transmission of cholera (13). Their main argument is because cholera is transmitted *via* multiple pathways, and intervention on one of them may not be enough to cut down the transmission. This is because individual interventions can have a different level of effectiveness, depending on the context. Despite the fact that the current sanitation ladder does not include waste treatment, they argued that the households that climbed the ladder from open defecation to improved sanitation have a reasonable epidemiological foundation. Considering this in our study, we were nevertheless able to find a significant association between the level of water coverage and sanitation and cholera transmission. With this statement, we however do not mean to imply that these are the only factors that explain and contribute to the containment (or outbreak) of cholera.

With the above discussion in mind, we must underline possible limitations of this study, starting from the cross-sectional nature of the data collection process. Indeed, the data were collected within a fixed time frame as a result of the cholera outbreak and therefore cannot provide a more general overview of the phenomenon as, for example, a longitudinal study would. Secondly, given the complexity of the hurdle model, which requires a considerable number of parameters to estimate, the sample size of 125 villages (for a population of 135,680 persons) is not exactly large, thereby relying more on the asymptotic approximation of the test statistics in the model (and possibly affecting the power of the analysis). Despite this, we still observed significant factors affecting the response variable. This is even more evident when including the outlier village of Kabobi (which reported an excessive prevalence of cholera compared to the others). However, note that due to data limitations on the field, we have not tried all possible combinations of models, so the inclusion of other variables remains a plausibility.

Some disparities were observed regarding the size of villages and their water and sanitation coverage. In fact, villages in Safo's Commune have an average of 2,144 inhabitants per village, while villages in Gabi and Sarkin Yamma are significantly smaller, with 807 inhabitants on average per village. More importantly, the villages without access to safe water are some of the smallest villages with an average of 450 inhabitants. Regarding water access, 87% (13 over 15) of the villages with more than 2,000 inhabitants have full water coverage. On the contrary, with regard to sanitation, the biggest villages are the ones with the poorest sanitation coverage. In fact, villages that reached full CLTS coverage have an average population of 752 inhabitants. Those facts can be explained by the fact that the CLTS approach is recommended globally for villages with fewer than 1,500 inhabitants for good implementation (bigger villages can be targeted by the CLTS approach but will be split into suburbs). Moreover, for water supply access, development partners and government tend to prioritize the biggest villages to target a higher number of beneficiaries and deliver a more sizable impact. Finally, before targeting villages with the CLTS approach, access to water is considered a selection criterion for the village prior to any sanitation project. This will result in an underrepresentation number of villages without water but involved in completed or partially completed CLTS projects (*n* = 10).

Nevertheless, findings were proven significant, and villages studied have similar features (cultural, socio-economical, or environmental factors), which limit the influence of other factors other than their water and sanitation coverage. The main differences observed and considered between villages are environmental factors such as the presence of surface water (lake, rivers) often in lower lands, which also influence land use. Some environmental factors were considered in the study; however, we should not exclude the possibility of other factors (collective immunity, local beliefs, etc.), which could have influenced our findings but were not captured in our study.

The findings suggest that significant access to safe water and sanitation at the village level offer a strong barrier against cholera. Hence, if aiming for the elimination of cholera, findings show that partial improved water access could be enough to limit cholera; however, for a stronger impact, full sanitation and hygiene coverage are recommended, as the presence of these facilities shows more significant influence than water access on cholera. This is totally complementary to all other known factors such surveillance (early detection and reporting) and infection prevention and control, treatment of cases, and vaccination, which are just as important in the prevention and control of cholera.

One of the limitations to achieve that objective will be the sustainability of CLTS services. If the area studied was targeted recently by CLTS projects, a 2016 study on CLTS in Niger (UNICEF) shows that 5 years after ODF certification, 39% of households in ODF villages reported reversion to open defecation. In that case, CLTS and water coverages are certainly not the only factor influencing the burden of cholera, as sustainability of those WASH services is probably the key to long-term impact on cholera.

Keeping in mind the limitations of our observational and cross-sectional study, we found a strong and significant association between improved access to water and sanitation (or both) to reduce the cholera transmission during the 2018 outbreak in Madarounfa. It also shed light on the need for more studies on this specific subject with more controlled and large-scale data collection schemes. This will undoubtedly strengthen the knowledge on cholera elimination, but more than that, it will help produce more tailored recommendations for each context to improve the effectiveness of the measures to be taken to reduce cholera transmission.

Overall, the study confirms recent findings mentioning that targeting cholera in key hotspots is an efficient approach and that sanitation, hygiene, and better water access have potential to significantly reduce the likelihood of cholera.

## Data Availability Statement

The original contributions presented in the study are included in the article/Supplementary Material, further inquiries can be directed to the corresponding author/s.

## Author Contributions

JG and GC contributed to the conceptualization and funding acquisition. JG and AK contributed to data curation. JG, MR, RM, and GC contributed to the formal analysis. JG, MR, AK, RM, and GC contributed to the investigation, writing, review, and editing. JG, MR, and RM contributed to the methodology. JG contributed to the project administration. All authors contributed to the article and approved the submitted version.

## Conflict of Interest

The authors declare that the research was conducted in the absence of any commercial or financial relationships that could be construed as a potential conflict of interest.

## Footnotes

^1^https://www.plateformecholera.info/index.php/bonus-page-2/national-strategies-plans/niger
