# Supplementary material for: Influence of Community-Led Total Sanitation and Water Coverages in the Control of Cholera in Madarounfa, Niger (2018)
Source: Front Public Health. 2021 Apr 29;9:643079. doi: 10.3389/fpubh.2021.643079 (PMC8118121; doi:10.3389/fpubh.2021.643079)
Supplement: Supplementary file 1 [file Data_Sheet_1.docx]

Supplementary Material

# Supplementary Material 1

**Analysis without Village Kabobi**

A close examination of the data reveals that one village, Kabobi, has a significantly high prevalence of cholera (10.8%) as 2018’s outbreak hit this small village strongly. Thus, to avoid overspecifying the effects of the variables, we ran an analysis while excluding this village. Full results are found in the table below.

Fully-improved and partial sanitation show significant contribution to the likelihood of cholera cases compared to having no access to improved sanitation at all. The likelihood of cholera cases decreases by 91%, with full sanitation access (p<0.01). Partial sanitation access decreases the likelihood by 78% (p<0.05). ). Water access, full and partial, is not significant to cholera cases at this level. None of the non-WASH factors are significant.

Where there are actual cholera cases, water access, in partial or full access levels, does not affect the cases of cholera at all significance levels. Meanwhile, full access to sanitation decreases the cases by a factor of 0.3, significant at a 95% confidence level (p<0.05). Non-WASH factors considered in our models remain non-significant (Suppl. Mat. 1).Non-WASH factors are also statistically equivalent to zero. The results hold when running WASH-only and non-WASH-only specifications.

*Table S1: Full and partial (WASH and non-WASH) hurdle models without Village Kabobi*

| Variables | Model  (1) | | Model  (2) | |
| --- | --- | --- | --- | --- |
|  | Coefficients | Transformed^#^ | Coefficients | Transformed^#^ |
| **Count Model** |  |  |  |  |
| Water Access - Partial | -0.225  (0.582) | 0.798 | -0.162  (0.619) | 0.239 |
| Water Access - Full | -0.595  (0.540) | 0.552 | -0.490  (0.562) | 0.183 |
| Sanitation – Partial | 0.543  (0.397) | 1.722 | 0.574  (0.411) | 1.683 |
| Sanitation – Full | -1.057*  (0.482) | 0.347* | -0.890  (0.504) | 0.089 |
| Distance to Water | 0.00000784  (0.0000288) | 1.000 |  |  |
| Distance to Contaminated Village | 0.0000396  (NA) | 1.000 |  |  |
| Road Access | 0.455  (0.357) | 1.576 |  |  |
| Log Theta | 0.087  (0.357) |  | 0.045  (0.225) |  |
| Intercept | 4.031***  (0.540) | 56.291*** | 4.260***  (0.564) | 258.485*** |
|  |  |  |  |  |
| **Zero Hurdle Model** |  |  |  |  |
| Water Access - Partial | 1.260  (0.684) | 0.779 | 1.200  (0.665) | 0.193 |
| Water Access - Full | 1.400*  (0.668) | 0.802 | 1.304*  (0.636) | 0.155* |
| Sanitation – Partial | -1.285*  (0.601) | 0.217* | -1.428*  (0.567) | 0.627* |
| Sanitation – Full | -2.278***  (0.635) | 0.093*** | -2.505***  (0.521) | 0.175*** |
| Distance to Water | -0.00004  (0.000167) | 0.499 |  |  |
| Distance to Contaminated Village | -0.0000842  (0.000213) | 0.499 |  |  |
| Road Access | 0.0242  (0.515) | 0.499 |  |  |
| Intercept | 0.02006  (0.691) | 0.505 | -0.352  (0.522) | 0.996 |

Standard errors in parentheses. *** p<0.0001, ** p<0.01, , * p<0.05

#Zero-part coefficients uses of logit link function; plogis() function was applied to transform the coefficients. The count-part coefficients were transformed via exponentiation.
